# Evaluation of the inhibitory effect of quercetin on the pharmacokinetics of tucatinib in rats by a novel UPLC–MS/MS assay

**DOI:** 10.1080/13880209.2022.2048862

**Published:** 2022-03-15

**Authors:** Ying Zhang, Ya-nan Liu, Saili Xie, Xuegu Xu, Ren-ai Xu

**Affiliations:** a Department of Pharmacy, HwaMei Hospital, University of Chinese Academy of Sciences (Ningbo No. 2 Hospital), Ningbo, China; b Ningbo Institute of Life and Health Industry, University of Chinese Academy of Sciences, Ningbo, China; c The First Affiliated Hospital of Wenzhou Medical University, Wenzhou, China; d The Eye Hospital of Wenzhou Medical University, Wenzhou, China

**Keywords:** Drug–drug interaction, cancer treatment, HER2-positive breast cancer

## Abstract

**Context:**

Tucatinib (CYP2C8 substrate) and quercetin (CYP2C8 inhibitor) are two common drugs for the treatment of cancer. However, the effect of quercetin on the metabolism of tucatinib remains unknown.

**Objective:**

We validated a sensitive method to quantify tucatinib levels in rat plasma based on ultra-performance liquid chromatography–tandem mass spectrometry (UPLC–MS/MS), which was successfully employed to explore the effect of quercetin on tucatinib pharmacokinetics in rats.

**Materials and methods:**

An Acquity UPLC BEH C18 column was applied to achieve the separation of tucatinib and internal standard (IS) talazoparib after protein precipitation with acetonitrile. Then, we used this assay to investigate the effect of different doses of quercetin (25, 50 and 100 mg/kg) on the exposure of orally administered tucatinib (30 mg/kg) in 24 Sprague-Dawley (SD) rats, which were randomly divided into three quercetin pre-treated groups and one control group (*n* = 6).

**Results:**

Our developed assay was verified in all aspects of bioanalytical method validation, involving lower limit of quantification (LLOQ), selectivity, accuracy and precision, calibration curve, extraction recovery, matrix effect and stability. After pre-treatment with 100 mg/kg quercetin, AUC_0→_*_t_*, AUC_0→∞_ and *C*_max_ of tucatinib were remarkably increased by 75.4%, 75.8% and 59.1% (*p* < 0.05), respectively, while CLz/F was decreased significantly by 47.3% (*p* < 0.05) when compared with oral administration of 30 mg/kg tucatinib alone. This change is dose-dependent.

**Conclusions:**

This study will help better understand the pharmacokinetic properties of tucatinib with concurrent use with quercetin, and more clinical verifications were inspired to confirm whether this interaction has clinical significance in humans.

## Introduction

**Figure 1.**
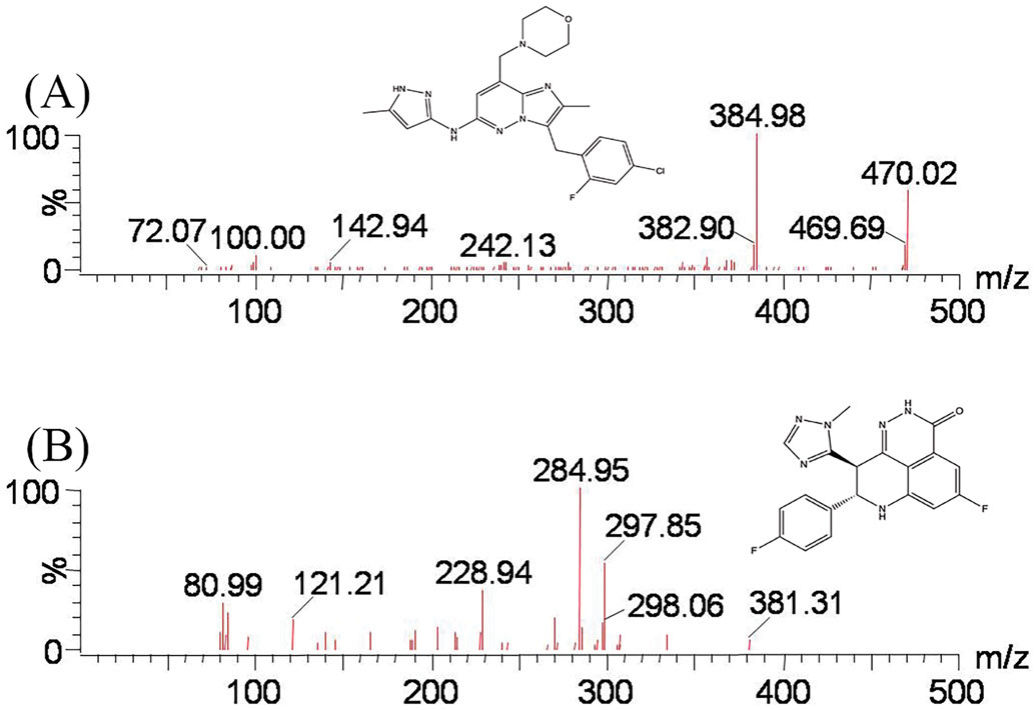
Mass spectra of tucatinib (A) and talazoparib (B), the IS for this study.

Tucatinib (Figure 1(A)) is an oral tyrosine kinase inhibitor (TKI) highly selective for the kinase domain of human epidermal growth factor receptor 2 (HER2), with minimal inhibition of epidermal growth factor receptor (Moulder et al. 2017). It was developed for the treatment of HER2-positive solid tumours, including breast cancer (BC) and colorectal cancer (Cesca et al. 2020). On April 2020, a marketing authorization for tucatinib for the treatment of adult patients with HER2-positive locally advanced or metastatic BC was granted by US Food and Drug Administration (FDA) (Lee 2020; Shah et al. 2021).

Following oral administration after a high-fat meal, area under the plasma concentration–time curve from time zero extrapolated to infinite (AUC_0→∞_) of tucatinib increases 1.5-fold and the time to peak concentration (*T*_max_) is delayed from 1.5 h to 4 h (Kunte et al. 2020). Given that cancer patients often take multiple drugs, whether the combination of tucatinib and other drugs would cause drug–drug interactions (DDIs) must be explored. CYP2C8 was proved to be the major metabolic enzyme *in vitro*, in addition to CYP3A to a lesser extent (Lee 2020). It was reported that gemfibrozil (a strong CYP2C8 inhibitor) increased the exposure of tucatinib when they were given with concomitant administration (Lee 2020). Thus, it is essential to establish a quantitative method for tucatinib in biological fluids to investigate its pharmacokinetic characteristics and potential DDIs. To the best of our knowledge, there is no published analytical and bioanalytical method available for tucatinib determination in biological fluids.

Quercetin is one of the flavonoids which are natural polyphenolic compounds found in numerous components of the human daily diet, including red wine, onions, apples, tea and grapefruit juice (Formica and Regelson 1995). It exerts a broad range of fascinating clinical properties, such as anticancer (Yin et al. 2021), anti-inflammatory (Li et al. 2016), antimicrobial (Heinz et al. 2010) and antiallergic (Mlcek et al. 2016) activities. Quercetin has the potential to inhibit cytochrome P450 (CYP), especially CYP2C8, and subsequent studies have demonstrated that it significantly inhibits the metabolism of CYP2C8 substrates (Kajosaari et al. 2005; Kim et al. 2005; Cao et al. 2017). However, the effect of quercetin on the metabolism of tucatinib remains unknown.

Therefore, the present study establishes a stable, simple and hypersensitive ultra-performance liquid chromatography–tandem mass spectrometry (UPLC–MS/MS) assay to detect plasma tucatinib concentration in rats. Moreover, our newly developed method explores the impact of diverse doses of quercetin on tucatinib exposure and its pharmacokinetic alterations in the experiment of rats.

## Materials and methods

### Materials and chemicals

Tucatinib, quercetin (the purity of each compound >98%) and talazoparib [internal standard (IS), purity >99%, Figure 1(B)] purchased from Beijing Sunflower Technology Development Co., Ltd. (Beijing, China) were employed in this study. Both acetonitrile and methanol were LC grade, and were supplied from Merck Company (Darmstadt, Germany). A Water Purification System from Milli-Q (Millipore, Bedford, MA) was used to acquire ultrapure water.

### Experimentation on animals

Sprague-Dawley (SD) male rats, weighing 190 ± 10 g, were purchased from Laboratory Animal Center, Wenzhou Medical University (Wenzhou, China). The rats were kept under appropriate environment, including proper humidity, temperature, light conditions, rodent diet and water. Before starting the experiment, all the rats were domesticated for 14 days under laboratory conditions to minimize their suffering. The animal studies were approved by the Animal Protection and Use Committee of Wenzhou Medical University (ID number: wydw2020-0111).

Tucatinib and quercetin were all prepared with carboxymethyl cellulose sodium (CMC-Na) solution with the concentration of 0.5%. After 12 h of fasting but with free access to water, we randomly divided 24 rats into four groups (*n* = 6): 0.5% CMC-Na (group A), quercetin (25 mg/kg, group B), quercetin (50 mg/kg, group C) and quercetin (100 mg/kg, group D). Tucatinib (30 mg/kg) was orally administered half an hour later. A series of blood samples of about 0.3 mL were then got from the tail veins of the rats at the time points of 0, 0.333, 0.667, 1, 1.5, 2, 3, 4, 6, 8, 12, 24 and 48 h, and were placed into 1.5 mL heparinized polythene tubes. Blood was then centrifuged at 4000×*g* at room temperature for 10 min, and the volume of 100 µL plasma was collected immediately and kept at −80 °C for further assay.

### Experimental conditions

In this experiment, a Waters Xevo TQ-S triple quadrupole tandem mass spectrometer (Milford, MA), connecting with a Waters ACQUITY UPLC I-Class system (Milford, MA) was used to operate the assays. Before analysis, we optimized and confirmed the mass spectrometric parameters: desolvation gas 1000 L/h, capillary voltage 2.0 kV, cone gas 150 L/h, desolvation temperature 600 °C and collision gas 0.15 mL/min. The autosampler for analysing all samples was kept at 10 °C, along with 40 °C for the temperature of the column. We implemented the measurement in positive ion mode using multiple reaction monitoring (MRM) with an electrospray ionization source (ESI). The specific parameters for tucatinib and IS are listed in Table 1. We manipulated data using the Quanlynx program with Masslynx 4.1 software (Milford, MA).

**Table 1.** Specific mass spectrometric parameters and retention times (RTs) for tucatinib and IS, including cone voltage (CV), and collision energy (CE).

Analyte	Precursor ion	Production	CV (V)	CE (eV)	RT (min)
Tucatinib	470.02	384.98	30	30	0.74
IS	381.31	284.95	30	25	0.73

A C18 column from Acquity UPLC BEH (2.1 mm × 50 mm, 1.7 μm) was employed to separate tucatinib and IS in the plasma. The mobile phase had two components including acetonitrile (solution A) and water possessing 0.1% formic acid (solution B). Linear gradient scheme was set and conducted as following: solution A was initially kept at 30%, increased from 30% to 85% in the following 0.9 min, then decreased from 85% to 30% in 0.1 min and kept stably at 30% for 1.0 min. The volume of each injection was 2.0 µL at a flow rate of 0.40 mL/min. The total run time was 2.0 min.

### Calibration curve and quality control samples

The compound was prepared with a proper amount of methanol to get tucatinib or IS stock solution with a 1.00 mg/mL concentration. Then, quality control (QC) samples and calibration curve were respectively prepared by the corresponding working solutions diluted with methanol from the stock solution of tucatinib. Each working solution with the volume of 10 µL was pipetted into blank plasma with the volume of 90 µL to obtain the sample for analysis. Finally, we acquired both the calibration curve standards and QC samples for tucatinib with the concentration levels of 0.5, 1, 5, 10, 50, 100, 200 and 400 ng/mL, along with 1, 80 and 320 ng/mL, respectively. The IS working solution for using in this study was 200 ng/mL by diluting with methanol. All prepared reagents were kept at −80 °C for further use.

### Sample treatment

Protein precipitation was performed by adding 300 µL acetonitrile to 100 µL of plasma, followed by a 20 µL IS working solution. The mixture was then centrifuged for 10 min at 13,000 rpm at the temperature of 4 °C after mixing for 2.0 min. Finally, 100 µL supernatant of each sample was drawn into a sample vial, where only 2.0 µL of the sample was used to inject into the autosampler for analysis.

### Bioanalytical method validation

We conducted a series of confirmatory experiments following the principles of FDA based on the validation of bioanalytical assay (Wang et al. 2021; Zhou et al. 2021): the lower limit of quantification (LLOQ), selectivity, calibration curve, precision and accuracy, recovery rate, stability and matrix effect.

### Statistical analysis

Origin 9.0 software (Originlab Company, Northampton, MA) in this study was applied to determine the average concentration versus time profile of tucatinib in plasma. The main pharmacokinetic parameters of tucatinib fitted with a non-compartmental model were calculated using DAS software (Drug and Statistics, Version 2.0, Shanghai University of Traditional Chinese Medicine, Shanghai, China). Pharmacokinetic parameters between groups were compared through one-way analysis of variance coupled with Dunnett's test using Statistical Package for the Social Sciences (version 17.0; SPSS Inc., Chicago, IL). *p* < 0.05 is statistically significant.

## Results

### Method validation

#### Selectivity and carry-over

**Figure 2.**
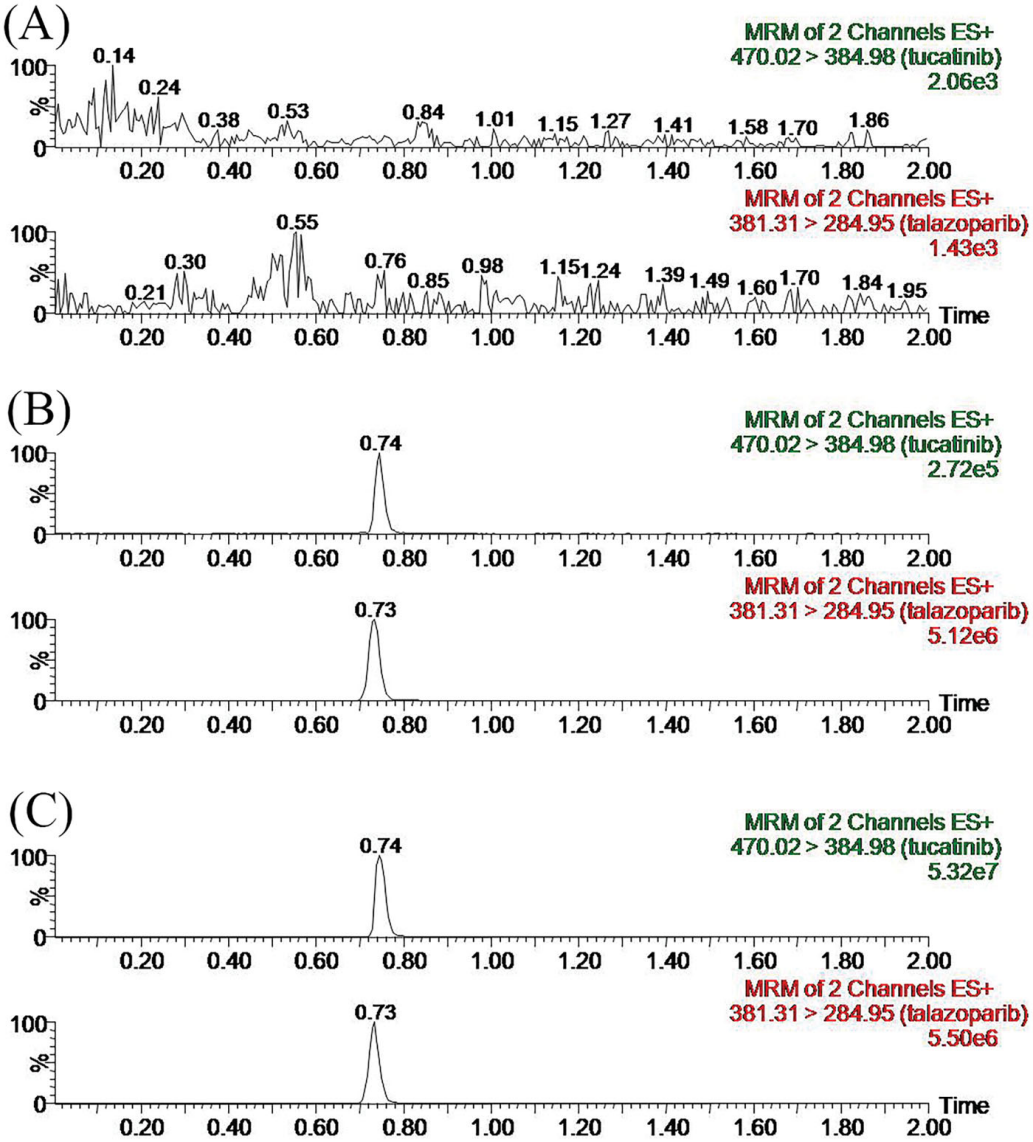
Representative chromatograms of tucatinib and IS in rat plasma: (A) blank plasma; (B) blank plasma spiked with standard solution at LLOQ (0.5 ng/mL) and IS; (C) sample obtained from a rat at 1.0 h after oral administration of 30 mg/kg tucatinib.

Figure 2 exhibits the representative MRM chromatogram peaks of six batches of blank rat plasma samples, the rat blank plasma sample pointed with the concentration of 0.5 ng/mL tucatinib at LLOQ and IS, along with the actual plasma sample after the oral administration of 30 mg/kg tucatinib in rats. Tucatinib and IS were identified at the retention periods of about 0.74 min and 0.73 min, respectively, without detectable endogenous interference. Consequently, the method has good selectivity and specificity to determine tucatinib in plasma. In addition, no carry-over was observed for either analyte or IS in rat plasma, since there was no interference peak detected following the injection of upper limit of quantification (ULOQ) samples.

#### Standard curve and LLOQ

The standard curve provided perfect linearity within the scope of 0.5–400 ng/mL for tucatinib in rat plasma. The typical linear regression formula of tucatinib was obtained as follows: *Y* = 1.59031×*X* + 0.790294 (*r*^2^=0.9976). The LLOQ was 0.5 ng/mL, possessing enough precision and accuracy (Table 2). Therefore, this method is sensitive to determine the plasma concentration of tucatinib in rats.

**Table 2.** The precision and accuracy of tucatinib in rat plasma (*n* = 6).

Analyte	Concentration (ng/mL)	Intra-day		Inter-day	
		RSD%	RE%	RSD%	RE%
Tucatinib	0.5	11.1	4.5	12.6	5.9
	1	7.0	8.8	7.3	9.3
	80	4.5	3.0	4.9	4.9
	320	3.5	–2.4	4.3	–5.0

#### Accuracy and precision

The accuracy and precision of tucatinib for inter-day and intra-day were quantified at LLOQ and three different QC concentrations on three separate days (*n* = 6), and the summary are presented in Table 2. The RE range of the intra- and inter-assay accuracy was −5.0% to 9.3%, with the RSD of the precision <12.6%. These outcomes indicated that the well-established assay had good accuracy, precision and reproducibility and could be applied for the quantitative analysis of tucatinib in the plasma samples from rats.

#### Matrix effect and extraction recovery

Table 3 summarizes the extraction recovery and matrix effect of tucatinib. The recovery rate of tucatinib was from 90.1% to 96.0% at three QC concentrations in rat plasma, and the recovery of IS was 93.3%, indicating few significant losses of both tucatinib and IS during the extraction process. Similarly, the matrix effects ranged from 92.3% to 101.6%, and the matrix effect of IS was 97.8%. The IS-normalized matrix factor of tucatinib had an RSD of 6.0% or less, which met the acceptance criteria (not more than 15%), manifesting that the matrix effect had no significant influence on rat plasma.

**Table 3.** Recovery and matrix effect of tucatinib in rat plasma (*n* = 6).

Analyte	Concentration (ng/mL)	Recovery (%)		Matrix effect (%)	
		Mean ± SD	RSD (%)	Mean ± SD	RSD (%)
Tucatinib	1	90.1 ± 3.6	4.0	101.6 ± 9.5	9.4
	80	93.7 ± 4.4	4.7	92.3 ± 3.9	4.2
	320	96.0 ± 5.3	5.5	99.9 ± 2.9	2.9

#### Stability

Stability experiments conducted in plasma samples at QC levels from Table 4 showed that tucatinib was durable and stable at ambient temperature for at least 3 h (short term stability), in the autosampler (10 °C) for 6 h thereafter extraction, three complete processes of freeze–thaw (–80 °C to ambient temperature), and also at −80 °C within four weeks (long-term stability).

**Table 4.** Stability results of tucatinib in plasma under different conditions (*n* = 5).

Analyte	Added (ng/mL)	Room temperature, 3 h		Autosampler 10 °C, 6 h		Three freeze–thaw		–80 °C, 4 weeks	
		RSD (%)	RE (%)	RSD (%)	RE (%)	RSD (%)	RE (%)	RSD (%)	RE (%)
Tucatinib	1	6.5	13.2	11.1	9.1	9.1	13.9	12.6	7.5
	80	4.8	4.3	4.8	4.9	7.2	5.0	4.9	5.3
	320	3.9	–8.4	2.8	–6.6	4.1	–6.8	4.0	–5.0

### Pharmacokinetics

**Figure 3.**
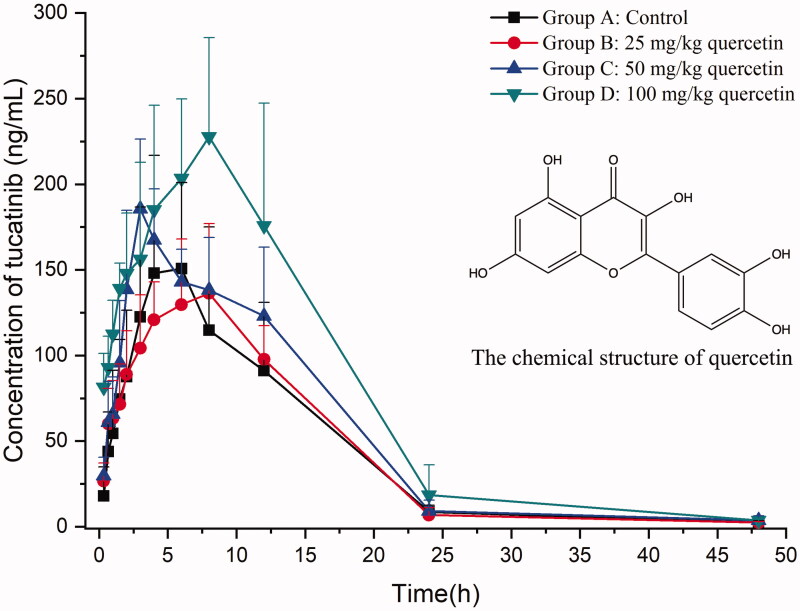
Mean plasma concentration–time curves of tucatinib in different treatment groups of rats. Group A: the control group (0.5% CMC-Na); group B: 25 mg/kg quercetin; group C: 50 mg/kg quercetin; group D: 100 mg/kg quercetin (*n* = 6, mean ± SD).

Using the novel developed bioanalytical assay based on UPLC–MS/MS technique, we detected the plasma concentrations of tucatinib in rats successfully, acquiring pharmacokinetics from different groups. Figure 3 exhibits the average concentration versus time curves of tucatinib in each rat group after taking 30 mg/kg tucatinib at a single oral dose. Table 5 sums up the essential pharmacokinetic parameters calculated under the mode of non-compartmental analysis.

**Table 5.** The main pharmacokinetic parameters of tucatinib in different treatment groups of rats.

Parameters	Group A	Group B	Group C	Group D
AUC_0→_*_t_* (ng/mL·h)	2049.70 ± 714.71	2046.83 ± 388.81	2540.31 ± 380.47*	3595.66 ± 797.53*
AUC_0→∞_ (ng/mL·h)	2066.04 ± 723.98	2067.67 ± 388.85	2566.64 ± 384.12*	3632.95 ± 792.89*
MRT_0→_*_t_* (h)	9.89 ± 0.74	9.65 ± 0.57	9.66 ± 1.31	10.06 ± 1.69
MRT_0→∞_ (h)	10.47 ± 0.98	10.21 ± 0.56	10.42 ± 1.66	10.59 ± 1.62
*t*_1/2_ (h)	6.37 ± 0.69	6.47 ± 1.31	6.71 ± 1.46	6.67 ± 0.85
*T*_max_ (h)	6.33 ± 2.94	6.80 ± 2.28	3.01 ± 0.63	7.17 ± 3.25
CLz/F (L/h)	16.32 ± 6.34	15.00 ± 3.32	11.90 ± 1.79*	8.60 ± 1.89*
*C*_max_ (ng/mL)	162.47 ± 48.91	149.64 ± 35.60	196.59 ± 54.15*	258.41 ± 42.73*

Group A: the control group (0.5% CMC-Na); group B: 25 mg/kg quercetin; group C: 50 mg/kg quercetin; group D: 100 mg/kg quercetin. (*n* = 6, mean ± SD).

Compared with group A, *p < 0.05.

## Discussion

The settings for UPLC–MS/MS were optimized for tucatinib and IS as to obtain maximal sensitivity, the product spectrums of tucatinib and IS are presented in Figure 1. In the method development, acetonitrile and water were first selected as the mobile phase. However, the supernatant of precipitated samples produced asymmetric peaks with poor sensitivity, which did not satisfy the method requirement. Therefore, 0.1% formic acid in water was adopted as mobile phase B. The optimized condition improved the ionization of the analyte and produced higher sensitivity and symmetric peaks.

A quick and straightforward extraction method is also essential due to the large number of plasma samples involved in pharmacokinetics or DDI-associated experiments (Wang et al. 2021; Zhou et al. 2021). In the present study, on the basis of the advantages of simplicity and rapidity, protein precipitation with acetonitrile was first assessed for sample preparation. The preliminary experiments indicated that acetonitrile method to precipitate proteins resulted in a higher recovery rate than other organic reagents, so we employed it.

Quercetin is a naturally occurring flavonoid and is mainly present as a glycoside in several components of the daily diet. It has been proved that quercetin is a potent CYP2C8 inhibitor and inhibits CYP2C8-catalysed metabolism *in vivo* (Kim et al. 2005). From the results of our study, when tucatinib was co-administered with 25 mg/kg quercetin in group B, the main pharmacokinetic parameters (AUC_0→_*_t_*, AUC_0→∞_, *t*_1/2_, *T*_max_, CLz/F and *C*_max_) of tucatinib had no significant differences compared with the control group A. However, compared with group A, groups C and D raised the AUC_0→_*_t_*, AUC_0→∞_ and *C*_max_ of tucatinib (*p* < 0.05), while decreased CLz/F (*p* < 0.05), indicating that the total tucatinib systemic exposure increased. Also, 100 mg/kg quercetin exhibited a more substantial inhibition on tucatinib metabolism than 50 mg/kg quercetin. Therefore, the concurrent use of tucatinib with high dose of quercetin should be treated with extreme caution. If their combined use is unavoidable, our data suggested dose reduction or interruption of tucatinib should be taken. Otherwise, the patient might suffer from some severe side effects (such as diarrhoea, palmar-plantar erythrodysesthesia syndrome and nausea) caused by increased tucatinib plasma levels (Murthy et al. 2020). The limitation of our research lies in the small number of rats used in the experiment.

## Conclusions

In the present experiment, we established a sensitive and accurate bioanalytical assay based on UPLC–MS/MS to determine tucatinib concentrations in plasma samples from rats. The optimized method had been carefully verified under FDA guidelines. In addition, we found that a high dose of quercetin exhibited inhibitory effect on the metabolism of tucatinib in rats. While considering the complex and varied clinical factors of cancer patients, further human clinical trials on tucatinib metabolism should be investigated to confirm the accuracy of the interaction and the significance.
